# Erythema Nodosum Leprosum in a Patient with Borderline Lepromatous Leprosy: A Case Report

**DOI:** 10.3390/idr17040083

**Published:** 2025-07-11

**Authors:** Guido Chiriboga, Qianyu Guo, Eric Zuberi, Harry Ross Powers, Libardo Rueda Prada

**Affiliations:** 1 Mayo Clinic Alix School of Medicine, Jacksonville, FL 32224, USA; 2 Department of Radiation Oncology, Mayo Clinic Florida, Jacksonville, FL 32224, USA; 3 Department of Internal Medicine, Mayo Clinic Florida, Jacksonville, FL 32224, USA; zuberi.eric@mayo.edu; 4 Department of Infectious Disease, Mayo Clinic Florida, Jacksonville, FL 32224, USA; 5 Department of Hospital Internal Medicine, Mayo Clinic Florida, Jacksonville, FL 32224, USA

**Keywords:** Mycobacterium Infections, Nontuberculous, *Mycobacterium leprae*, Leprosy, Leprosy, Lepromatous, Armadillos

## Abstract

Background: Leprosy, caused by *Mycobacterium leprae*, presents on a spectrum ranging from tuberculoid to lepromatous disease. Borderline lepromatous leprosy represents an unstable immunological state that predisposes patients to immune-mediated reactions, including erythema nodosum leprosum (ENL), a severe inflammatory complication. Case Presentation: We report a case of a 62-year-old female with borderline lepromatous leprosy who presented with recurrent facial cellulitis and later developed disseminated ENL. She was initially diagnosed following a series of facial infections and confirmatory skin biopsy. Months later, she developed systemic inflammatory lesions consistent with ENL, requiring hospitalization. She was treated with high-dose corticosteroids for ENL and methotrexate to treat type 1 reaction and continued multidrug therapy (MDT) with minocycline, rifampin, and clarithromycin for leprosy, which led to significant clinical improvement. Conclusion: This case highlights the diagnostic challenges of leprosy in the United States and the importance of recognizing ENL as a severe immunologic complication requiring prompt intervention. A multidisciplinary approach is essential for optimal patient outcomes.

## 1. Introduction

Leprosy is a chronic infection caused by *Mycobacterium leprae* or *Mycobacterium lepromatosis*, an acid-fast bacillus that primarily affects the skin and peripheral nerves. It presents along a spectrum, with tuberculoid (paucibacillary) leprosy representing a strong cell-mediated immune response and lepromatous (multibacillary) leprosy indicating immune tolerance to the bacilli. Approximately 30 to 50% of patients with leprosy present with immunologic reactions, mainly of two types. Type 1 (TR1) or reversal reactions more often occur in patients with borderline disease. It presents with abrupt inflammatory changes to the skin, nerves, or both, resulting in erythema, edema, and possible ulceration. TR1 is the leading cause of nerve damage in leprosy and may lead to disability, predominantly resulting in foot drop, wrist drop, and facial palsy [1,2]. Type 2 (T2R) or erythema nodosum leprosum (ENL) more often occurs in patients with lepromatous disease [1]. ENL is characterized by painful cutaneous nodules, systemic inflammation, and potential organ involvement. It most commonly occurs within one year of starting MDT and often presents with systemic symptoms like fever, anorexia, and malaise, as well as arthralgias and dactylitis [2].

Immunologic reactions in leprosy can be difficult to distinguish from other immune reactions and autoimmune diseases. Both T1R and T2R are clinically diagnosed, and no specific serum markers are available. Biopsy histology is a mainstay of diagnostic confirmation, but given the reactions’ rarity and diverse constellation of symptoms, many patients do not receive a prompt diagnosis without specialized care [1,2]. ENL, in particular, requires prompt recognition and treatment to prevent long-term complications, such as visual impairment, claw hand, foot drop, or lagophthalmos [3,4].

## 2. Case Presentation

A 62-year-old female with a two-year history of borderline lepromatous leprosy presented to Mayo Clinic Florida with recurrent facial cellulitis. She resides in an endemic region where armadillos are a primary reservoir of *Mycobacterium leprae* [5].

Her symptoms began approximately five years prior to this presentation, when she developed a rash on her left upper extremity that worsened with sun exposure. Over time, the rash progressed to her right upper extremity, forming plaques, and eventually spread to her face, chest, abdomen, and lower extremities. She also developed edema and numbness in the dorsal surfaces of her hands and feet, along with a persistent cold sensation in her nose. Despite evaluations by several dermatologists and multiple skin biopsies, no diagnosis was initially established.

Three years after symptom onset, she presented to Mayo Clinic Florida. In collaboration with the National Hansen’s Disease Clinical Center (which provides free consultation, follow-up, and medication administration for patients diagnosed with leprosy), she was diagnosed with borderline lepromatous leprosy. A skin biopsy showed diffuse interstitial and cord-like granulomatous inflammation throughout the dermis and focally in the subcutis, with a foamy histiocyte appearance. Fite staining was strongly positive; AFB (Ziehl-Neelsen) and GMS stains were negative. Additional testing ruled out Sézary syndrome, blast cell skin infiltration, and clonal lymphoid expansion. She was started on methotrexate (15 mg weekly divided in doses 7.5 mg/day on days 1 and 2) and glucocorticoids (prednisone: 60 mg/day for 7 days, 40 mg/day for 7 days, 20 mg/day for 7 days; then 5 mg daily) to proactively manage reversal reaction followed by antibiotic therapy with rifampin, minocycline, and moxifloxacin. Due to joint pain, moxifloxacin was later switched to clarithromycin. This regimen led to significant improvement in her dermatologic symptoms.

Seven months after her diagnosis, she began tapering off steroids. Eleven months after her diagnosis, she developed her first episode of facial cellulitis involving the left temple and forehead, marked by erythema, swelling, pain, and leukocytosis following a wasp sting. A second episode occurred one month later—twelve months after diagnosis—also triggered by a wasp sting. Both episodes resolved with intravenous antibiotics.

**Figure 1:**
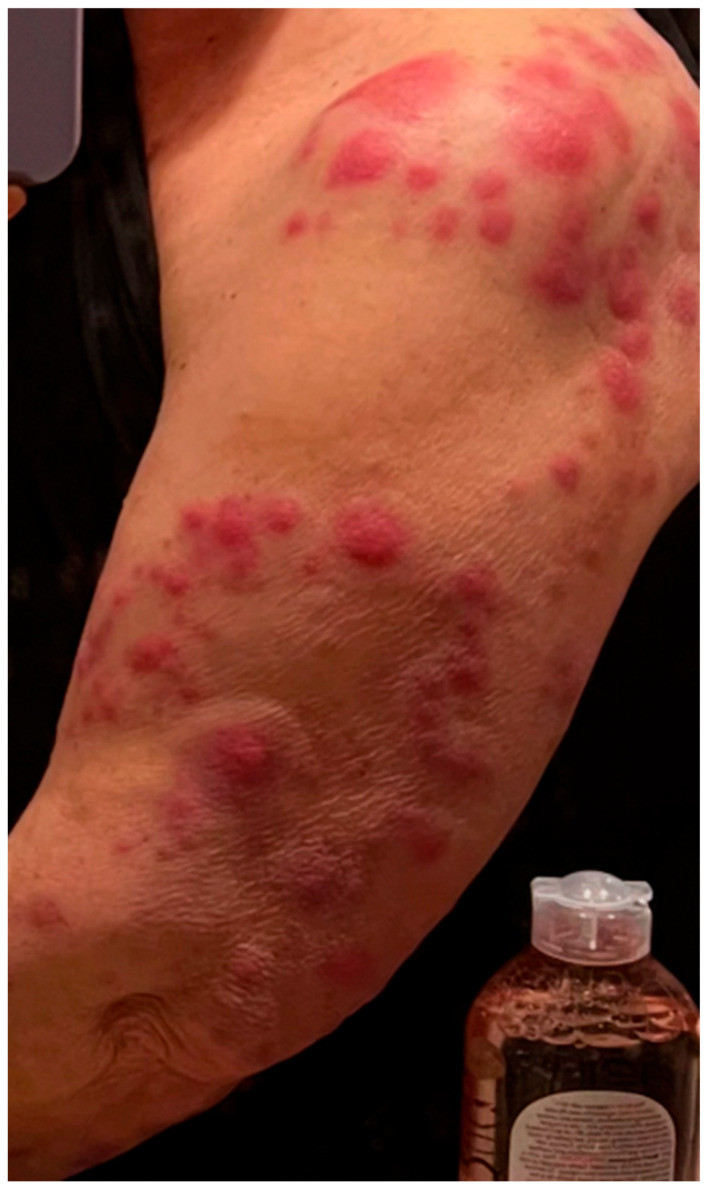
Multiple skin lesions involving the left upper extremity.

**Figure 2:**
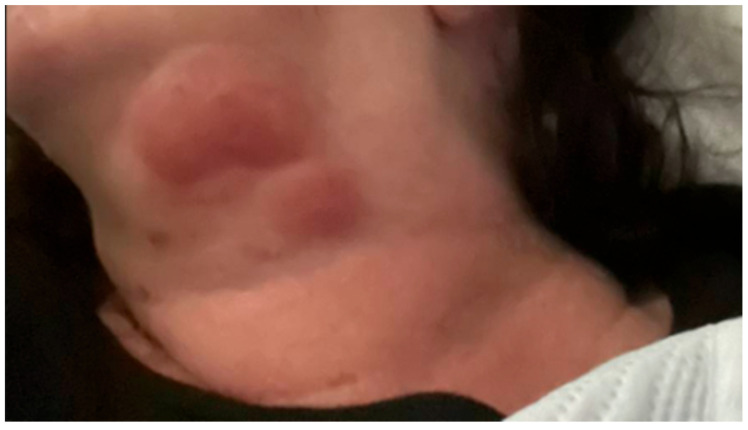
Large, indurated lesion on the left anterior triangle of the neck.

Eighteen months after her diagnosis, the patient developed widespread erythema and dozens of new lesions involving the left upper extremity (Figure 1), back, abdomen, and bilateral lower extremities, extending to the distal thighs. She also developed a large, indurated lesion on the left anterior triangle of the neck (Figure 2), which was mildly tender but not accompanied by fever or chills. She was admitted for management of ENL.

On admission, her vital signs were notable for tachycardia with a heart rate of 121. Laboratory studies revealed white blood cell count 21.8 × 10^9^/L (reference range 3.4–9.6 × 10^9^/L), hemoglobin 15 g/dL (11.6–15.0 g/dL), ESR 80 mm/h (0–29 mm/h), C-reactive protein 106 mg/L (<5 mg/L), liver aminotransferases AST 53 U/L (8–43 U/L), ALT 53 U/L (7–45 U/L), and alkaline phosphatase 102 U/L (35–104 U/L).

She was treated with intravenous methylprednisolone (125 mg every eight hours for three days), which was then tapered to twice daily for two days before transitioning to oral prednisone (40 mg daily) on hospital day five. The patient showed significant clinical improvement in her ENL lesions with corticosteroid therapy. However, she experienced steroid-induced insomnia and developed reactive leukocytosis and thrombocytosis, both of which improved with steroid tapering. Inflammatory markers, including CRP, trended downward.

She was discharged on prednisone 40 mg daily with a plan to taper once having transitioned to thalidomide, along with methotrexate 7.5 mg weekly, minocycline 100 mg daily, and a once-a-month combination of rifampin and clarithromycin. She continues to follow up with our Infectious Disease clinic and the National Hansen’s Disease Center in Baton Rouge, Louisiana.

## 3. Discussion

The patient’s presentation with non-specific diffuse plaque lesions highlights one of the challenges of leprosy diagnosis—its ability to mimic other dermatologic and infectious conditions. In this case, a high index of suspicion and confirmatory biopsy were essential for establishing the correct diagnosis. Furthermore, her episodes of ‘recurrent cellulitis’ were likely type 1 reversal reactions. The initial pathologic findings included diffuse granulomatous inflammation with foamy histiocytes and positive Fite staining. The patient’s two-year history of leprosy symptoms prior to diagnosis at Mayo Clinic Florida, despite visits with multiple specialists and undergoing biopsies, further highlights the need to increase awareness of the disease. Prompt diagnosis and treatment of ENL can ameliorate or prevent long-term sequelae, including disability [3,4]. This is especially true in areas with higher incidence, like Central Florida, which now accounts for 81% of cases in the state and almost one fifth of cases nationally [5,6].

The development of ENL months after the initial diagnosis is a well-documented phenomenon, occurring in 30 to 50% of patients with lepromatous or borderline lepromatous leprosy. ENL is driven by an exaggerated immune response to dead or dying *Mycobacterium leprae* bacilli, often triggered by other infections, stress, or initiation of MDT [2]. Our patient’s condition necessitated high-dose corticosteroids with transition to thalidomide as an outpatient, which remain a mainstay of treatment for severe ENL. Methotrexate was introduced to proactively manage type 1 reaction, as recommended by the Hansen Center [7,8].

MDT is essential for eradicating *Mycobacterium leprae* and preventing disease progression. Our patient was treated with minocycline, rifampin, and moxifloxacin (having later transitioned to clarithromycin due to intolerance with joint pain), the regimen recommended by the Hansen Center [9]. In the United States, the alternative dapsone-based MDT regimen includes rifampin and clofazimine. Clofazimine is classified as an investigational drug and is not used for acute type 2 reactions (TR2) but may be used for chronic TR2 in patients unable to take thalidomide or high-dose steroids [10]. Regular follow-up with a specialized leprosy center is crucial for monitoring treatment response and detecting potential complications [2,8].

This case highlights the importance of early recognition of leprosy and its associated immune reactions. Despite the 1991 WHO goal for disease “elimination” (incidence of <1 per 10,000), there were 219,075 cases reported in 2011, with India and Brazil reporting the most cases. Notably, under the current treatment paradigm, disease prevalence has often been below incidence year-to-year [11]. In the United States, leprosy incidence peaked in 1983 and decreased until 2000, when a gradual increase in reports began. In 2020, 159 new cases were reported, approximately 20% in Florida. Of those, 81% were reported in Central Florida, with a higher prevalence of locally acquired disease than in past decades. Zoonotic transmission from armadillos as a reservoir has been implicated [5,6]. Clinicians should maintain an awareness of leprosy’s varied presentations and its potential complications, particularly in patients residing in endemic areas.

## 4. Conclusions

This case illustrates the diagnostic challenges and management considerations in borderline lepromatous leprosy complicated by ENL. Early recognition and a multidisciplinary treatment approach, including corticosteroids and immunosuppressive therapy, are essential for favorable outcomes. Ongoing follow-up is crucial to monitor for disease progression and treatment complications. Increased awareness of leprosy in endemic areas remains vital for timely diagnosis and intervention.

## Data Availability

No new data were created or analyzed in this study. Data sharing is not applicable to this article.
